# Sentence-Level Provenance for AI Medical Record Summarization in a Click-to-Inspect Interface: Formative Usability Evaluation

**DOI:** 10.2196/95644

**Published:** 2026-09-08

**Authors:** Andrew Parambath, Giordana Pulpo, Vince Hartman

**Affiliations:** 1 Stanford Medicine, 900 Welch Road, Suite 350, Palo Alto, CA, 94304, United States, 1 2672979144; 2 Abstractive Health, New York, NY, United States

**Keywords:** medical informatics, usability testing, clinical decision support systems, artificial intelligence, AI, natural language processing, electronic health records, human-centered design, health IT, trust calibration

## Abstract

**Background:**

Large language models can generate fluent summaries of longitudinal medical records, but in high-stakes clinical settings, verification burden remains a barrier to trust. Existing provenance mechanisms such as document-level citations and section references often require manual search within long, fragmented notes, limiting their usefulness during time-constrained workflows for clinicians.

**Objective:**

This study aimed to design and evaluate a sentence-level provenance interface (“click to inspect”) that enables rapid verification of AI-generated longitudinal medical record summaries at the level of individual statements.

**Methods:**

Between November 2023 and January 2024, we conducted a formative usability study using remotely moderated usability sessions via Zoom to evaluate a web-based sentence-level provenance interface for AI-generated longitudinal medical record summaries. A convenience sample of clinicians was recruited through email outreach to academic and professional networks across the United States. Formative usability testing was conducted with 46 clinician interactions using synthetic longitudinal patient charts. Participants included medical students, residents, and attending physicians across multiple specialties, including internal medicine, dermatology, radiology, plastic surgery, anesthesiology, interventional radiology, obstetrics and gynecology, and family medicine. Usability was assessed using the System Usability Scale and net promoter score, alongside qualitative feedback.

**Results:**

Clinicians reported high usability (mean System Usability Scale score 86.25, SD 7.77; 95% CI 83.96‐88.54 from 46 participants) and a positive overall experience (net promoter score of 35; 22/46, 47.8% promoters; 18/46, 39.1% passives; and 6/46, 13% detractors). Participants described rapid access to supporting evidence as critical for trust calibration during first-pass chart review. Qualitative feedback identified friction in traditional citation-based interfaces and supported sentence-level inspectability as a low-friction verification mechanism.

**Conclusions:**

Sentence-level provenance transforms AI-generated summaries from static narratives into interactive verification tools. An approach that enables rapid, selective inspection of individual claims during longitudinal chart review may reduce verification burden and support calibrated reliance in high-risk clinical contexts.

## Introduction

### Background

Large language models can generate fluent summaries of long clinical text, yet in high-risk domains such as health care, trust depends not only on accuracy but also on verifiability [1,2]. This is especially true for longitudinal medical record summaries, which condense years of fragmented documentation into a single narrative [3]. Unlike encounter summaries written immediately after care [4,5], longitudinal summaries often serve as a clinician’s first exposure to an unfamiliar patient’s history.

Without prior mental models, clinicians may struggle to distinguish rare but true details from model error and may find it difficult to trust these outputs [6,7]. The burden of verification in time-constrained environments associated with locating supporting evidence within long and redundant notes remains a central barrier to adoption [2,8].

Most deployed clinical summarization systems have focused on encounter-based summaries such as visit notes, discharge summaries, and emergency department documentation, which are generated immediately after a clinical interaction [4,5,9,10]. In these settings, clinicians already possess a mental model of the patient from direct participation in care, and summaries primarily support recall, documentation, and communication [11]. In contrast, longitudinal medical record summaries synthesize years of fragmented documentation across multiple encounters and specialties into a single narrative [3,12]. This creates a distinct cognitive challenge because clinicians must determine whether individual summarized statements are supported by the underlying medical record while simultaneously constructing an understanding of the patient’s history. As a result, longitudinal summarization requires not only accurate summaries but also efficient mechanisms for verifying individual clinical claims during first-pass review [2,8].

### Prior Work

Existing provenance approaches commonly rely on document-level citations, section references, or phrase-level highlights [9-12]. While familiar from academic writing, these mechanisms often require manual search within cited documents. In longitudinal clinical documentation, relevant evidence may be embedded within multipage notes, making targeted verification inefficient.

Recent AI-assisted clinical information retrieval and summarization systems, including Perplexity, OpenEvidence, and Microsoft Copilot, have incorporated provenance through document-level citations, section references, or highlighted supporting snippets to improve transparency [13-15]. Similarly, human-centered explainable AI research has emphasized that explanations should be designed to support user decision-making and integrate naturally into existing workflows rather than simply increasing model transparency [16,17]. However, even when provenance is available, clinicians must still navigate lengthy clinical notes and determine whether the cited evidence adequately supports individual summarized claims, a process that can interrupt reading flow and increase cognitive burden during time-constrained chart review [18-21].

Although these approaches improve transparency, little work has specifically examined provenance interfaces designed to support rapid verification of individual clinical statements during longitudinal chart review. This represents an important gap in the literature because clinicians reviewing unfamiliar patients must efficiently distinguish supported clinical information from potential model errors while simultaneously building an understanding of the patient’s history, yet few provenance interfaces have been designed specifically to support this verification workflow.

### Study Objectives

We designed and evaluated a sentence-level provenance interaction model (“click to inspect”) that makes every summary sentence directly inspectable in its source context. We conducted formative usability testing to assess whether this interaction reduces friction during verification and supports calibrated trust.

The objective of this study was to evaluate the usability of a sentence-level provenance interface for AI-generated longitudinal medical record summaries. Specifically, we aimed to (1) evaluate the perceived usability of the interface using the System Usability Scale (SUS); (2) assess clinicians’ overall experience using the net promoter score (NPS); and (3) characterize clinician perceptions of verification workflow, verification burden, and trust through qualitative usability interviews. Collectively, these aims sought to determine whether sentence-level provenance represents a usable and practical approach for supporting rapid verification of AI-generated longitudinal medical record summaries during clinical chart review.

## Methods

### Overview of Study Design

This study was a formative usability evaluation of a web-based sentence-level provenance interface for AI-generated longitudinal medical record summaries. The system was evaluated using synthetic patient charts designed to simulate common longitudinal documentation patterns encountered in clinical practice.

The purpose of this study was to evaluate the usability of the sentence-level provenance interface rather than the accuracy or clinical performance of the underlying summarization system. The AI-generated summaries used during testing were iteratively refined through structured clinician feedback to ensure that they reflected realistic longitudinal clinical documentation and supported evaluation of the verification workflow. Accordingly, the summaries served as a vehicle for evaluating the provenance interface and were not used to assess the performance of a specific language model or summarization approach.

### The Tool and Interface

**Figure 1.**
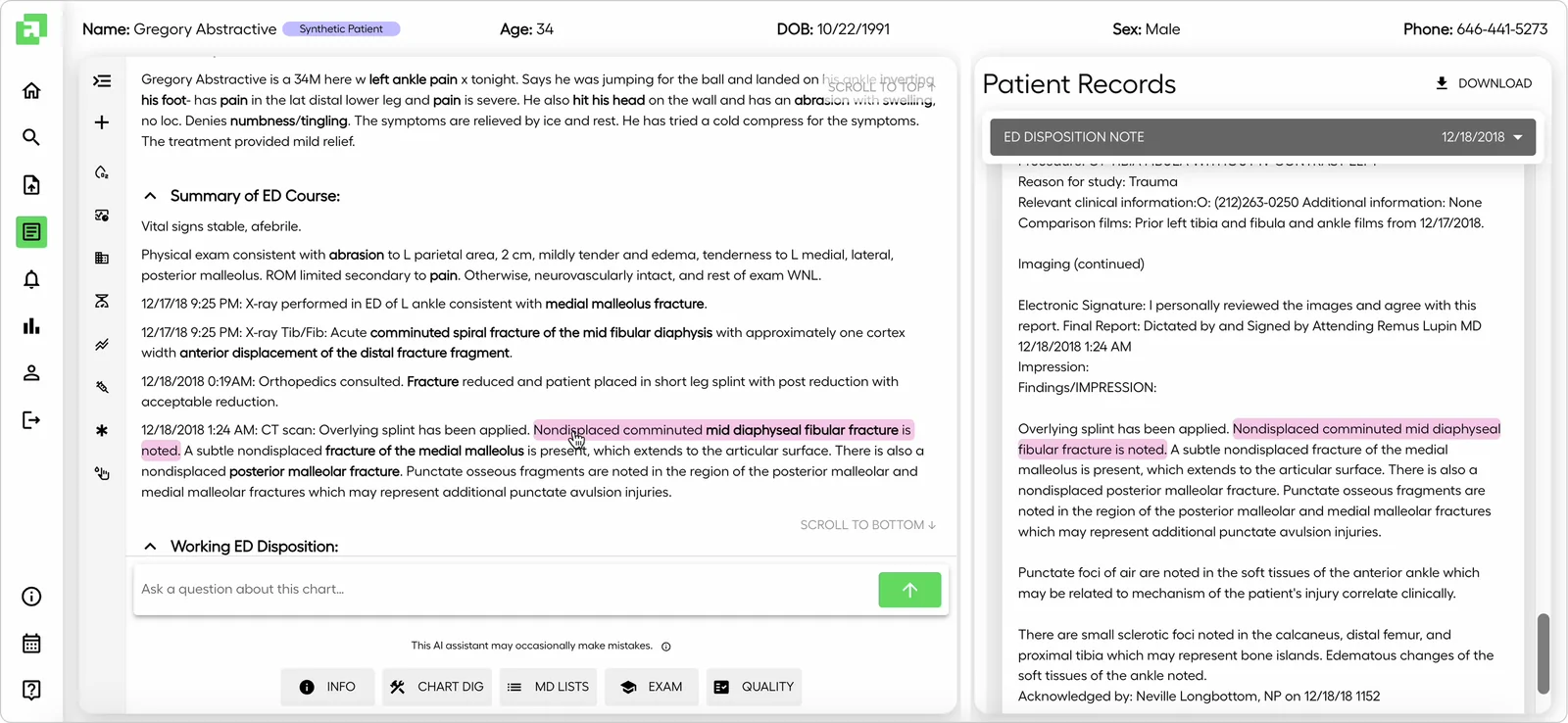
Click-to-inspect summaries in use. A clinician clicks on a sentence in the longitudinal AI summary (left), and the system opens the originating note (right), automatically scrolling to and highlighting the matched supporting sentence in context.

**Figure 2.**
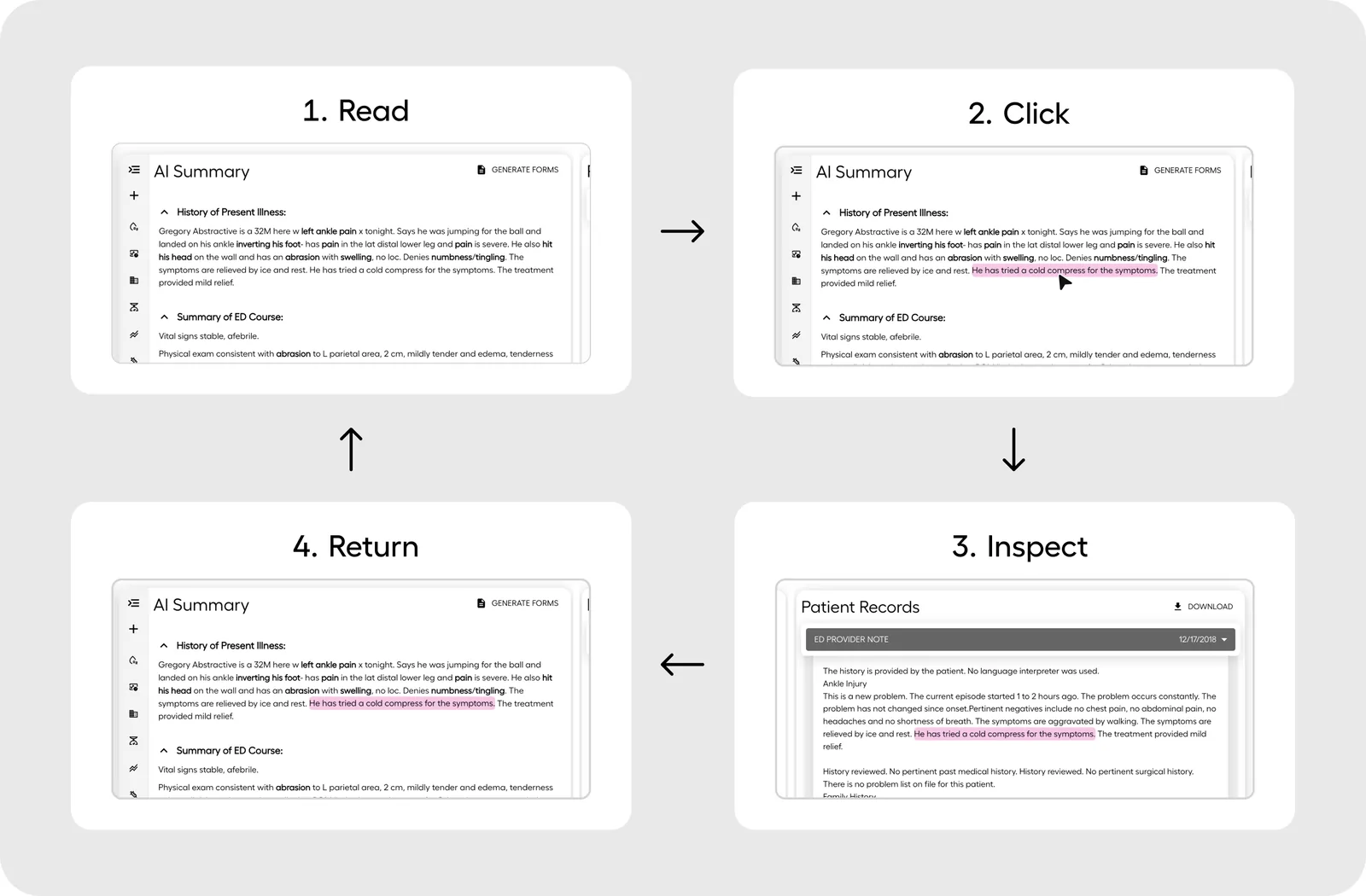
The click-to-inspect loop, where selecting a summary sentence opens the originating note, scrolls to the matched supporting sentence, and highlights it in context for rapid verification and return to reading.

**Figure 3.**
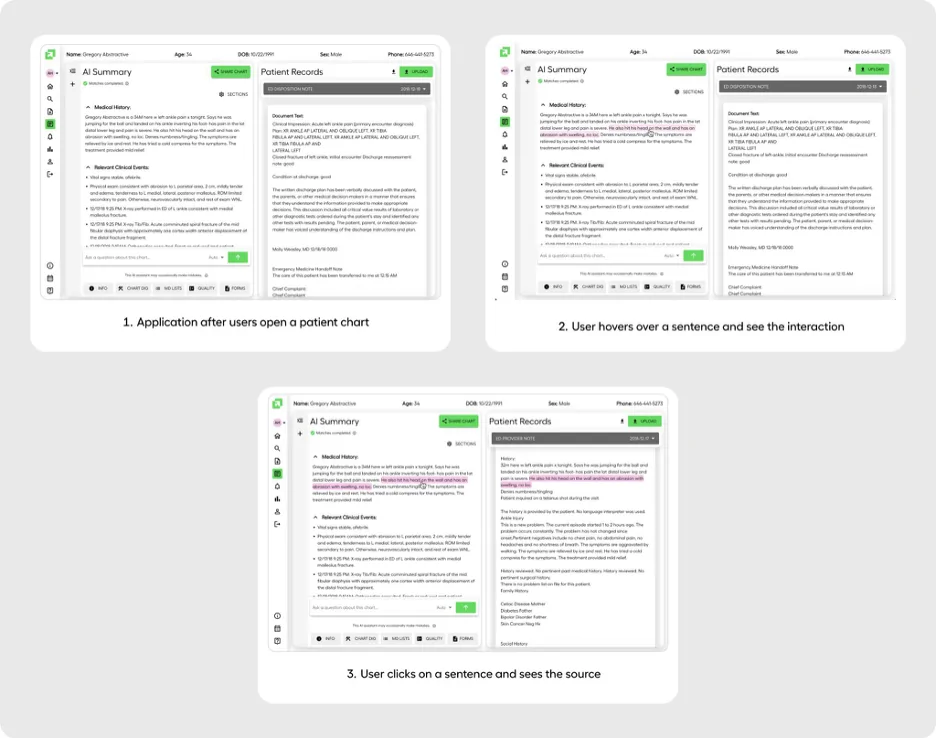
The general overview of the workflow that a user would go through when navigating the application.

Participants interacted with a web-based sentence-level provenance interface presenting AI-generated longitudinal medical record summaries alongside a source note viewer. Every sentence within the summary was interactive. When a participant selected a summary sentence, the interface opened the originating clinical note in a side-by-side view, automatically scrolled to the corresponding source sentence, and highlighted the matched text in context. This interaction was designed to support a rapid verification workflow by allowing clinicians to inspect supporting evidence without manually searching through source documentation (Figures 1-3).

Two synthetic patient charts were developed for this study. The first represented an acute emergency department encounter involving an ankle injury, diagnostic imaging, and specialty consultation. The second represented a longitudinal outpatient history spanning multiple years of multispecialty documentation. Both charts were designed to reflect common longitudinal documentation patterns encountered in clinical practice, including fragmented histories, cross-specialty documentation, and varying levels of clinical complexity. Synthetic charts were authored and reviewed by board-certified clinicians to ensure clinical realism while avoiding the use of real patient information.

Sentence-level provenance links were generated using embedding-based semantic similarity between summary sentences and candidate source note sentences. Sentence embeddings were generated using the transformer-based sentence embedding model all-mpnet-base-v2 (Hugging Face). Before embedding, source clinical notes were converted from their structured representation into plain text and segmented into sentence-level units. Text was initially partitioned using line breaks, runs of 2 or more white space characters, bullet symbols, and dash space separators. Each resulting text segment was trimmed of surrounding white space and further segmented into individual sentences using the Natural Language Toolkit (Team NLTK) Punkt sentence tokenizer. Empty segments were discarded. No abbreviation expansion, spelling correction, or clinical terminology normalization was performed before embedding.

Cosine similarity was calculated between each summary sentence and all candidate sentences within the originating clinical note. For each summary sentence, the highest-scoring source sentence was selected as the provenance reference. A cosine similarity threshold of 0.70 was used to categorize provenance matches. Summary sentences whose highest similarity score was 0.70 or more were categorized as verified, whereas those with scores below 0.70 were categorized as unverified while remaining clickable within the interface. The threshold was established during iterative internal clinical review of provenance quality to identify a value that consistently distinguished clinically meaningful sentence matches from weaker semantic associations. This represented a clinician-informed calibration process rather than a formally validated statistical threshold.

The provenance interface used a one-to-one sentence mapping. Each summary sentence was linked to a single originating clinical note and a single highest-scoring source sentence within that note. The underlying summarization pipeline preserved the originating note associated with each generated summary sentence and did not generate individual summary sentences by combining information across multiple source notes. Although a summary sentence could synthesize information from multiple sentences within the same note, the interface intentionally displayed only the highest-scoring supporting sentence. This design was selected to provide a single, consistent point of verification while minimizing visual complexity during chart review. Findings from the usability evaluation supported this approach as participants consistently described a single salient provenance location as intuitive and sufficient for verification during longitudinal chart review.

### Participant Sampling and Recruiting

Clinicians were recruited between November 2023 and January 2024 through email outreach to medical students, resident physicians, and attending physicians using academic institutional mailing lists, residency program listservs, and professional networks across multiple health systems in the United States. Recruitment emails described the study as a usability evaluation of a clinical summarization interface and invited interested participants to complete a brief interest form. Participation was voluntary. Eligible participants were required to be actively engaged in clinical training or practice. No additional exclusion criteria were applied.

### Usability Testing Procedures

Moderated usability sessions were conducted remotely via Zoom (Zoom Video Communications) between November 2023 and January 2024. Each session lasted approximately 30 minutes and followed a semistructured usability interview guide to ensure consistency across participants (Multimedia Appendix 1). The guide consisted of standardized clinical scenarios; predefined task-based usability assessments; and open-ended interview questions designed to evaluate clinician workflow, verification behavior, and overall usability of the sentence-level provenance interface.

During each session, participants interacted with the web-based interface while reviewing AI-generated longitudinal patient summaries. Participants were encouraged to use a think-aloud protocol, verbalizing their reasoning as they reviewed summaries and selectively inspected supporting source material. Moderators used nondirective follow-up prompts to clarify why participants chose to verify particular statements and how the interface influenced their verification process while allowing for flexibility to explore emerging usability issues. Participants were not required to inspect a predetermined number of statements, allowing for observation of natural verification behavior during clinical chart review. All sessions were audio and video recorded and transcribed for subsequent qualitative analysis.

### Data Collection and Analysis

Following interaction with the interface, participants completed the SUS and an NPS questionnaire. The SUS is a validated 10-item instrument measuring perceived usability on a scale from 0 to 100. The NPS was assessed using the standard likelihood to recommend question (0-10), with respondents categorized as promoters (9-10), passives (7-8), and detractors (0-6).

In addition to quantitative usability measures, qualitative data were collected during these remotely moderated usability testing sessions via Zoom using a think-aloud session, in which participants described their verification strategies, perceived workflow friction, trust in AI-generated summaries, and overall experience using the interface during the Zoom session.

Descriptive statistics were calculated for quantitative usability outcomes. Mean SUS scores, SDs, and 95% CIs were calculated across participants. The NPS was calculated using the standard formula of the percentage of promoters minus the percentage of detractors.

Audio recordings were transcribed and independently reviewed by 2 researchers. Qualitative data were analyzed using an inductive thematic analysis approach to identify recurring themes related to verification workflow, verification burden, trust calibration, and integration into clinical practice. Themes were iteratively refined through discussion until consensus was achieved.

### Ethical Considerations

This study was reviewed and approved by Pearl IRB (Indianapolis, Indiana, United States; 2025-0079) under an expedited review process and was determined to involve minimal risk. All study procedures were conducted in accordance with the approved protocol. Participants provided verbal informed consent before taking part. To protect participant privacy and confidentiality, all usability sessions were audio and video recorded solely for research purposes, securely stored in accordance with the approved study protocol, and analyzed by authorized study personnel. Synthetic patient charts were used throughout the study to ensure that no real patient information or protected health information was accessed or disclosed during usability testing. Participants received a US $25 electronic gift card upon completion of the usability session.

## Results

Recruitment invitations were distributed through multiple overlapping institutional mailing lists, residency program listservs, and professional networks. As a result, the exact number of clinicians who received the invitation could not be determined because individual recipients may have received invitations through more than one distribution channel. During the recruitment period, 107 clinicians expressed interest in participating in the study, of whom 46 (43%) completed a moderated usability session and were included in the final analysis (Table 1).

**Table 1.** Characteristics of study participants (N=46).

Characteristics	Values
Age range (y)	20‐60
Clinical specialty groups represented, n (%)	
Internal medicine	11 (23.9)
Surgical specialties	10 (21.7)
Radiology	4 (8.7)
Dermatology	3 (6.5)
Neurology	3 (6.5)
Ophthalmology	3 (6.5)
Emergency medicine	2 (4.3)
Family medicine	2 (4.3)
Obstetrics and gynecology	2 (4.3)
Pediatrics	2 (4.3)
Hematology and oncology	2 (4.3)
Anesthesiology	1 (2.2)
Psychiatry	1 (2.2)

Recruitment concluded when participant feedback demonstrated recurring and consistent observations regarding workflow, verification behavior, and overall usability. At that point, the sample size was considered sufficient for the formative usability evaluation because additional recruitment was unlikely to yield substantially new usability insights.

The mean SUS score was 86.25 (SD 7.77; 95% CI 83.96‐88.54), indicating high perceived usability of the click-to-inspect interface.

The NPS was 35, calculated using the standard method (percentage of promoters minus percentage of detractors on the 0‐10 NPS scale). Of the 46 participants, 22 (47.8%) were classified as promoters (scores of 9‐10), 18 (39.1%) were classified as passives (scores of 7‐8), and 6 (13%) were classified as detractors (scores of 0‐6).

During the moderated think-aloud sessions, participants frequently described rapid access to supporting evidence as critical to their willingness to rely on the summary during initial review. Clinicians reported that being able to verify individual statements with a single click reduced cognitive load compared with document-level citation systems that require manual searching within lengthy notes. Participants noted that traditional provenance mechanisms often identify the source document but do not eliminate the effort required to locate the exact supporting passage within that document.

Sentence-level inspectability was consistently described as predictable and low friction. Participants reported that the absence of persistent citation markers or confidence indicators preserved reading flow while maintaining immediate access to supporting evidence when needed. Participants also emphasized that the ability to selectively verify clinically important or unexpected statements aligned well with real-world chart review workflows.

Representative participant comments included the following:

Being able to click directly on the sentence and see the exact line in the note makes it much easier to trust the summary.

Normally I have to scroll through the whole note to find where something came from. This saves a lot of time.

I like that I can verify the statements that matter without breaking my reading flow.

## Discussion

### Principal Findings

This formative usability evaluation found that a sentence-level provenance interface for AI-generated longitudinal medical record summaries demonstrated high perceived usability among clinicians, with a mean SUS score of 86.25 (SD 7.77) and an NPS of 35. Consistent with the objectives of this study, clinicians reported that the click-to-inspect interaction reduced the effort required to verify AI-generated clinical statements while preserving reading flow during longitudinal chart review. Participants consistently described rapid access to supporting source material as enabling selective verification of clinically important statements without interrupting their workflow. These findings support the usability of sentence-level provenance as a verification interface independent of the underlying summarization model and suggest that embedding provenance directly within individual summary statements may reduce verification burden while supporting calibrated trust in AI-generated longitudinal summaries.

Our findings suggest that the value of provenance extends beyond simply identifying the source of summarized information. Participants reported that the interface made it easier to verify individual clinical statements while maintaining focus on understanding the patient’s history. Instead of navigating through lengthy clinical notes to locate supporting evidence, clinicians could inspect the source material only when they felt that additional verification was necessary. This workflow appeared to reduce the effort associated with verification and allowed clinicians to remain focused on interpreting the summary rather than searching for supporting documentation.

These findings are consistent with those of prior work in explainable AI showing that transparency mechanisms are most effective when they support user decision-making within existing workflows rather than simply exposing model outputs [16,17]. Existing AI-assisted clinical summarization and information retrieval systems commonly provide document-level citations, section references, or highlighted supporting snippets [13-15,22-25]. Although these approaches improve transparency, clinicians must still determine whether the cited material adequately supports the summarized clinical claim while navigating lengthy and fragmented documentation. Our findings suggest that sentence-level provenance may reduce this burden by making supporting evidence immediately accessible at the point where verification is needed.

The findings are also consistent with principles from human factors research. Clinical reasoning is typically performed at the level of individual findings, diagnoses, medication changes, and clinical events rather than entire documents. Participants consistently reported that they wanted the ability to verify only selected statements rather than every sentence within a summary. Clinicians naturally focused on information that was clinically important, unexpected, or likely to influence decision-making. The interface supported this workflow by allowing participants to selectively inspect supporting evidence without interrupting their review of the remainder of the summary. Participants also noted that the absence of persistent citation markers or confidence scores preserved reading flow while maintaining immediate access to supporting evidence when desired. Together, these observations suggest that minimizing visual complexity while preserving rapid inspectability may represent an important design principle for future AI-assisted clinical documentation systems.

Several limitations should be considered when interpreting these findings. First, this was a formative usability evaluation conducted using synthetic patient charts. Although synthetic data enable controlled testing without privacy concerns, they may not fully capture the variability, ambiguity, and complexity of real-world longitudinal clinical documentation. Second, although participants represented a broad range of clinical specialties, recruitment took place through academic institutional mailing lists, residency program listservs, and professional networks, which may limit the generalizability of these findings to other clinical settings. Third, this study evaluated perceived usability rather than objective performance. Outcomes such as time to verification, verification accuracy, detection of hallucinated or unsupported statements, and downstream effects on clinical decision-making were not measured. Accordingly, this study cannot determine whether sentence-level provenance improves clinicians’ ability to identify summarization errors or unsupported clinical claims. Finally, provenance mapping relied on embedding-based semantic similarity, and its robustness across heterogeneous clinical documentation warrants further evaluation.

Future studies should evaluate sentence-level provenance in real-world clinical environments using production electronic health record data and AI-generated longitudinal summaries. Important outcome measures include verification time, verification accuracy, detection of unsupported or hallucinated statements, changes in information-seeking behavior, and downstream effects on clinical decision-making. Comparative studies should also evaluate sentence-level provenance against conventional document navigation and document-level citation interfaces to determine whether it improves verification efficiency, clinical reasoning, and trust calibration. Additional work is needed to understand how alternative provenance presentation strategies, including confidence scores and prioritization cues, influence verification behavior and clinician reliance on AI-generated summaries.

This study suggests that sentence-level provenance is a feasible and well-accepted approach for supporting verification of AI-generated longitudinal medical record summaries. As generative AI becomes increasingly integrated into electronic health record workflows, the design of verification interfaces may become as important as improvements in model performance. Supporting efficient, low-friction verification has the potential to improve clinician confidence, promote appropriately calibrated trust, and facilitate the safe integration of AI-generated longitudinal summaries into routine clinical practice.

### Conclusions

Sentence-level provenance represents a human-centered approach to improving verifiability in AI-generated longitudinal medical record summaries. By making every summary sentence directly inspectable within its source context, the click-to-inspect interaction embeds verification into the act of reading rather than positioning it as an external search task. In formative usability testing, clinicians described the interface as intuitive and compatible with time-constrained workflows and reported high perceived usability. Although further empirical validation is needed, these findings suggest that designing provenance at the level of individual clinical statements may better align AI summarization tools with real-world clinical practice and support calibrated reliance in high-risk settings.

## Supplementary material

10.2196/95644 Multimedia Appendix 1 Interview guide for moderators of usability testing sessions.

## Abbreviations

NPS: net promoter score
SUS: System Usability Scale
