# Supplementary material for: Sentence-Level Provenance for AI Medical Record Summarization in a Click-to-Inspect Interface: Formative Usability Evaluation
Source: JMIR Hum Factors. 2026 Sep 8;13:e95644. doi: 10.2196/95644 (PMC13552831; doi:10.2196/95644)
Supplement: Supplementary file 1 [file humanfactors-v13-e95644-s001.pdf]

Usability Testing Guide

Overview

This guide standardizes the conduct of moderated remote usability sessions evaluating the Abstractive Health application. It is intended to ensure consistency across participants while allowing moderators the flexibility to explore participant reasoning and emerging usability issues. Participants use a think-aloud protocol throughout the session.

The application is opened in advance by the study team and presented to the participant through a shared screen. Participants are given remote control of the shared screen and interact with the interface directly throughout the session. Each participant is assigned one clinical scenario and then completes the standardized task sequence described below.

Session Format

Sessions are conducted remotely via Zoom and last approximately 30 minutes. The study team shares its screen with the application already open and grants the participant remote control, allowing the participant to navigate the interface directly while the moderator observes. A moderator facilitates each session, guides the participant through the assigned scenario and task sequence, and administers the closing questions. Sessions are recorded to support subsequent analysis; recording begins only after verbal consent is obtained.

Test Environment

All tasks are performed using synthetic patient records created for evaluation purposes. No protected health information is accessed or displayed at any point during the session. Synthetic records include multiple encounters and clinical note types across time so that longitudinal summarization can be meaningfully evaluated.

Participant Briefing

Before beginning, the moderator reviews the following points with each participant:

- The purpose of the session is to evaluate the usability of the application, not the performance or knowledge of the participant.
- There are no right or wrong answers. Any difficulty encountered during a task reflects the design of the interface rather than participant ability.
- Participants are asked to think aloud continuously, describing what they are looking at, what they expect, and why they choose a given action.
- Participants may ask questions at any time. The moderator may defer answering in order to observe how the participant would proceed independently.
- All patient information displayed during the session is synthetic.
- The session is recorded for analysis. Participants may decline recording, skip any question, or stop the session at any point.

Clinical Scenarios

Each participant completes one of the following representative clinical workflows:

Scenario A. Hospital Discharge Summary

Imagine you are preparing a hospital discharge summary and would like to review the patient's longitudinal clinical history using the AI-generated summary.

Scenario B. Emergency Department Evaluation

Imagine you are evaluating a patient arriving in the emergency department and want to rapidly review the patient's longitudinal medical history before making clinical decisions.

Scenario C. Emergency Department Documentation

Imagine you are preparing an emergency department provider note and would like to use the AI-generated summary to assist documentation.

Standardized Usability Tasks

Participants complete the following tasks while verbalizing their thought process. Tasks are presented in a fixed order, and moderators use the accompanying prompts only as needed to elicit participant reasoning.

Task 1. Initial Orientation to the Interface

- Take control of the shared screen and review the application as first displayed.
- Describe your first impressions of the interface.
- Describe what you expected to see.

Moderator prompts

– What do you think this page is showing?
– Is this what you expected?

Task 2. Understanding the AI-Generated Summary

The participant reviews the AI-generated longitudinal summary.

Moderator prompts

– What do you think “Summary Notes” represents?
– How would you use this summary during patient care?

Task 3. Navigating Summary Templates

The participant locates an alternative summary template and then returns to the general summary view.

Moderator prompts

– How would you switch to another summary template?
– Was this navigation what you expected?

Task 4. Verifying Supporting Evidence

The participant identifies the clinical note supporting a selected summary statement.

Moderator prompts

- What would you do if you wanted to verify this statement?
- Is this interaction what you expected?
- What do you think the highlighted text represents?
- What do you think of this verification process?

Task 5. Exploring Source Documentation

The participant locates additional source clinical notes used to generate the summary.

Moderator prompts

- Where would you expect to find additional supporting documentation?
- Was this workflow intuitive?

Task 6. Editing the Summary

The participant describes how they would modify the AI-generated summary if edits were needed.

Moderator prompts

- How would you make a change to this text?
- Is this what you expected?

Task 7. Exporting the Summary

The participant describes how they would transfer the completed summary into the electronic health record.

Moderator prompts

- What do you expect to happen?
- What do you see here?
- Is this workflow intuitive?

Task Difficulty Rating

Immediately following each task, the participant rates the difficulty of task completion on a scale from 1 (very easy) to 10 (very difficult) and explains the reasoning behind the rating.

Closing Interview Questions

The moderator asks the following open-ended questions:

1. What are your overall impressions of the product?
2. How would you describe your overall experience using the interface?
3. What aspects of the workflow worked particularly well?
4. What aspects of the workflow could be improved?
5. If you could change one feature of the interface, what would it be and why?
6. If this software were available in your clinical practice, how often would you expect to use it? Why?

Think-Aloud Protocol

Throughout the session, participants are encouraged to verbalize their reasoning while interacting with the interface. Moderators use nondirective prompts such as:

- Tell me what you're thinking.
- What are you looking for?
- Why did you choose that action?
- What did you expect would happen?
- Was anything confusing?
- What would you do next?

Post-Session Questionnaires

Following completion of the usability session, participants complete:

- System Usability Scale (SUS)
- Net Promoter Score (NPS)
